# Supplementary material for: Category-biased patches encircle core domain-general regions in the human lateral prefrontal cortex
Source: Neuropsychologia. Author manuscript; Available in PMC 2026 Jul 10. (PMC13353797; doi:10.1016/j.neuropsychologia.2025.109164)
Supplement: Multimedia component 1 [file NIHMS2186099-supplement-Multimedia_component_1.docx]

# Supplementary Figures

| **Supplementary Figure 1.**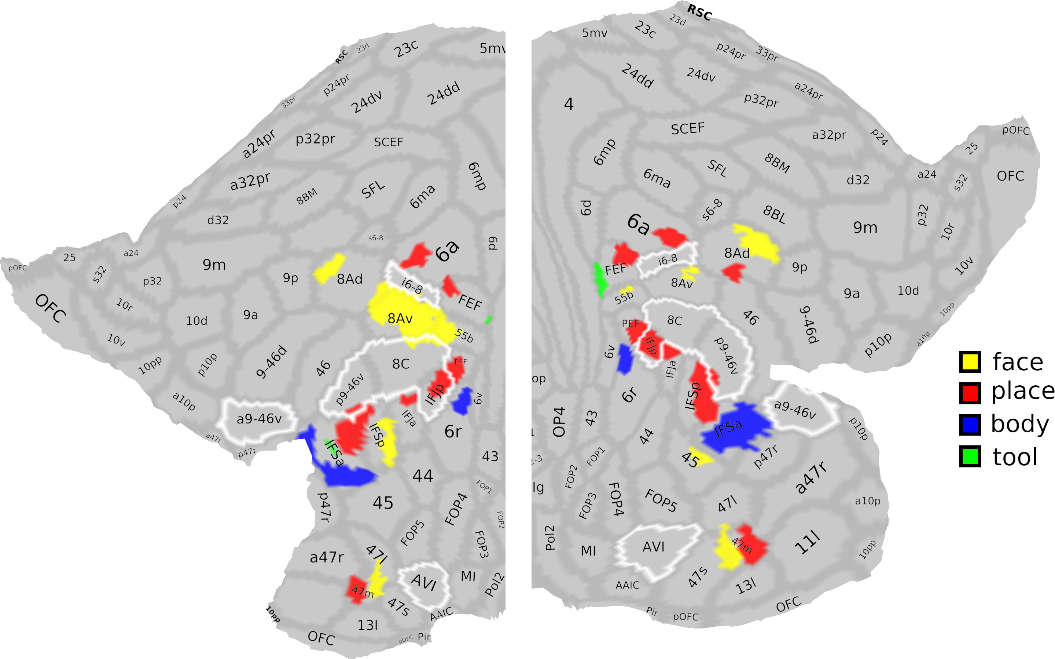Category-biased patches in the HCP dataset from **Figure 1** overlaid on the areal labels of the HCP MMP1.0 (Glasser et al., 2016).Data in this figure are available at <https://balsa.wustl.edu/3q0lx> |
| --- |

| 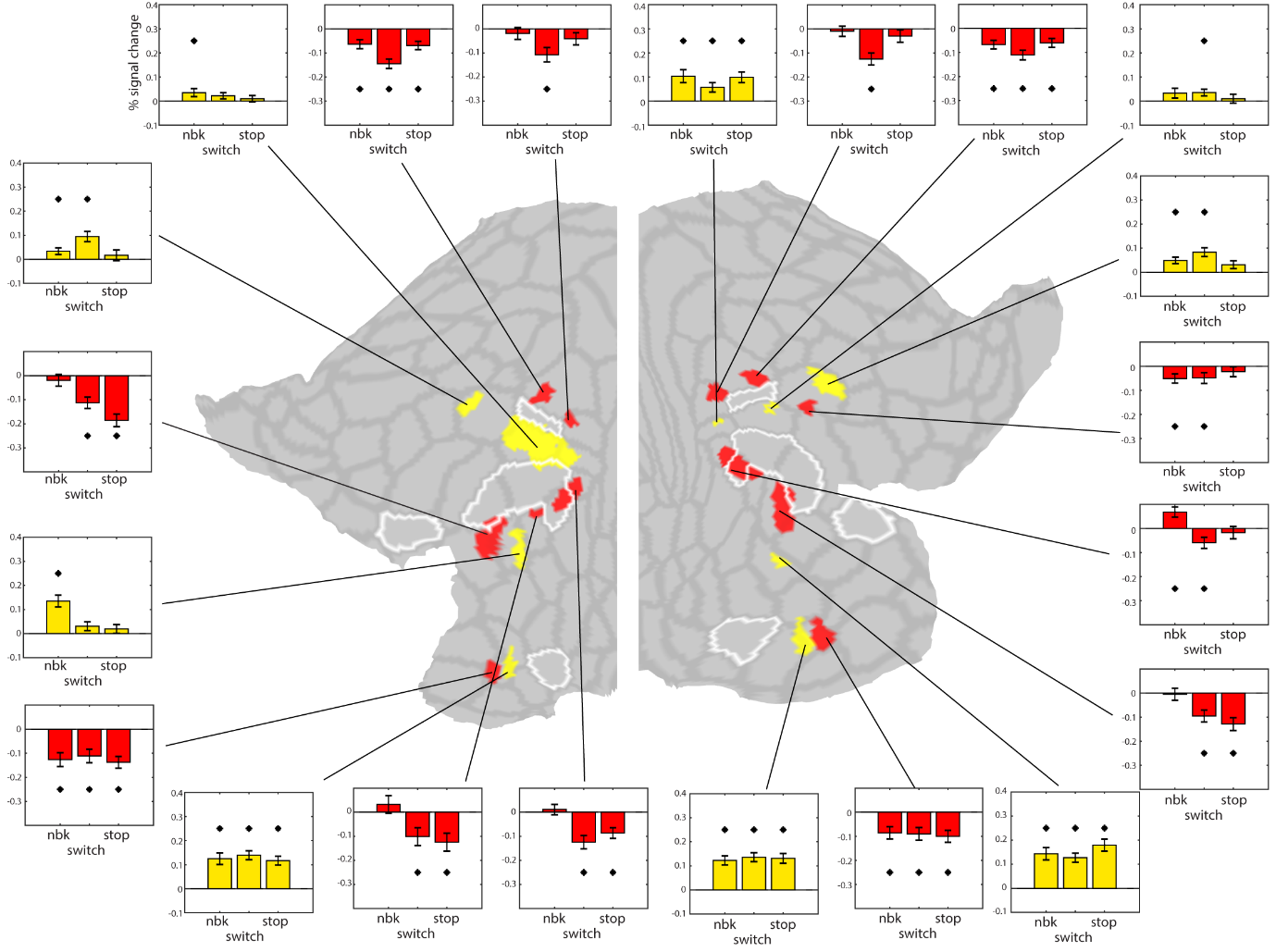**Supplementary Figure 2.** Estimated response (face minus place) for each EF task [n-back (nbk), switch, stop] to the HCP’s n-back defined face and place ROIs. Core MD regions (white borders) are based on the definition in (Assem et al., 2020). * indicates p<0.05 Bonferroni corrected within each patch for 3 tasks. |
| --- |
